# Estimating the risk of cardiovascular outcomes and all‐cause mortality in individuals with type 2 diabetes: Validation of the UKPDS outcomes model using TECOS and EXSCEL data

**DOI:** 10.1111/dom.70280

**Published:** 2025-11-12

**Authors:** Ruth L. Coleman, Amanda I. Adler, Philip M. Clarke, Darren K. McGuire, Rury R. Holman

**Affiliations:** ^1^ Diabetes Trials Unit, Radcliffe Department of Medicine University of Oxford Oxford UK; ^2^ Health Economics Research Centre, Nuffield Department of Population Health University of Oxford Oxford UK; ^3^ School of Population and Global Health University of Melbourne Melbourne Victoria Australia; ^4^ Division of Cardiology, Department of Medicine University of Texas Southwestern Medical Center and Parkland Health and Hospital System Dallas Texas USA

**Keywords:** cardiovascular risk estimates, mortality, myocardial infarction, type 2 diabetes, UKPDS outcomes model

## Abstract

**Aims:**

To evaluate United Kingdom Prospective Diabetes Study Outcomes Model version 2 (UKPDS‐OM2) cardiovascular risk estimates for people with type 2 diabetes using TECOS and EXSCEL data.

**Materials and methods:**

We compared model‐simulated and TECOS and EXSCEL observed event rates for the composite outcome of cardiovascular death, myocardial infarction (MI) or stroke, each component, and all‐cause mortality. Risk factors analysed were age, sex, race/ethnicity, height, diabetes duration, atrial fibrillation, albuminuria, and baseline plus annual measures of smoking, HDL‐cholesterol, LDL‐cholesterol, weight, systolic blood pressure, HbA_1c_, heart rate, white cell count, haemoglobin, and estimated glomerular filtration rate. Other factors were prior ischemic heart disease, heart failure, amputation, blindness, kidney failure, MI, stroke, and diabetic foot ulcer.

**Results:**

Median follow‐up was 3.0 and 3.2 years for the 14 671 TECOS and 14 752 EXSCEL participants, respectively. The primary outcome occurred in TECOS for 839 (11.4%) and 851 (11.6%) sitagliptin and placebo group participants, respectively (hazard ratio [HR] 0.98, 95%CI 0.89–1.08), compared with UKPDS‐OM2 simulated events of 776 (10.6%) and 778 (10.6%), respectively (relative risk 1.00). The primary outcome occurred in EXSCEL for 839 (11.4%) and 905 (12.2%) once‐weekly exenatide and placebo group participants, respectively (HR 0.92, 95%CI 0.84–1.01), compared with UKPDS‐OM2–simulated events of 579 (7.9%) and 593 (8.0%), respectively (relative risk 0.98).

**Conclusions:**

UKPDS‐OM2 accurately simulated relative risks between randomized groups in both trials. The proportion of participants with the primary outcome was accurately estimated in both TECOS arms but underestimated by around one‐third in both EXSCEL arms.

## 1 INTRODUCTION

People with type 2 diabetes, versus those without, are at greater risk of cardiovascular (CV) disease and premature death, particularly those who have prevalent atherosclerotic CV disease (ASCVD).^1^, ^2^ In 2008, the U.S. Food and Drug Administration enacted a requirement that new drugs and biologics for glycemic control in patients with type 2 diabetes needed to demonstrate CV safety.^3^ This has resulted in a large number of randomized controlled trials of new types of therapies involving people with type 2 diabetes.^4^ In recent years, there have been calls to extend these trials from being placebo‐controlled to having an active therapy comparator.^5^ The design of such trials of glycemic therapies in populations at high or low risk of CV disease needs to be informed by accurate predictions of risk for CV‐related and other clinical outcomes for people with type 2 diabetes.

Although many CV risk calculators are available, few are designed predominantly to estimate risk in populations without prior CV disease, such as SCORE‐2.^6^ There have been considerable efforts to formally evaluate a wide range of risk prediction tools for people with type 2 diabetes,^7^ but there has been much less focus on the applicability and validity in the context of clinical trial design. We sought to evaluate the performance of the previously published United Kingdom Prospective Diabetes Study Outcomes Model version 2.2 (UKPDS‐OM2),^8^ a type 2 diabetes patient‐level simulation tool that estimates lifetime outcomes. We used patient‐level data from two placebo‐controlled randomized trials in patients with type 2 diabetes, the Trial Evaluating Cardiovascular Outcomes with Sitagliptin (TECOS)^9^ and the EXenatide Study of Cardiovascular Event Lowering (EXSCEL).^10^ All participants in TECOS had ASCVD, whereas only 73.1% in EXSCEL had ASCVD.

Our objective was to evaluate the accuracy of UKPDS‐OM2 simulations of the primary composite CV outcome and its components in both trials. These components included CV death, all‐cause mortality (ACM), fatal or nonfatal myocardial infarction (MI), fatal or nonfatal stroke, hospitalization for heart failure (hHF), and hospitalization for acute coronary syndrome (hACS). Secondary objectives included evaluating model performance split by age groups, sex, or history of CV events (EXSCEL only).

## 2 MATERIALS AND METHODS

### 2.1 TECOS

TECOS^9^ was a noninferiority CV outcome safety trial evaluating the effect of sitagliptin treatment compared with placebo, added to usual care, on CV outcomes in patients with type 2 diabetes and ASCVD. Between December 2008 and July 2012, 14 671 patients were recruited from 38 countries and followed for a median of 3.0 years. The primary composite CV outcome was a 4‐point major adverse CV event (MACE‐4), namely the first adjudicated occurrence of CV death, nonfatal MI, nonfatal stroke, or hospitalization for unstable angina (hUA). The secondary composite outcome was a 3‐point major adverse CV event (MACE‐3), which did not include hUA. Secondary endpoints were CV death, fatal or nonfatal MI, fatal or nonfatal stroke, hUA, ACM, hHF, and the composite of hHF or CV death.

### 2.2 EXSCEL

EXSCEL^10^ was a CV outcome trial (superiority for efficacy and noninferiority for safety) evaluating the effect of once‐weekly subcutaneous exenatide (EQW) compared with placebo, added to usual care, in participants with type 2 diabetes, of whom 73.1% had prior ASCVD. A total of 14 752 participants were recruited from 35 countries between June 2010 and September 2015, and followed for a median of 3.2 years. The primary composite CV outcome was MACE‐3. Secondary endpoints were ACM, CV death, fatal or nonfatal MI, fatal MI, fatal or nonfatal stroke, fatal stroke, hHF, and hACS.

### 2.3 UKPDS‐OM2

We used version 2 of UKPDS‐OM2, which is a second‐generation lifetime simulation model for people with type 2 diabetes^8^ that was constructed using patient‐level data from the 30‐year UKPDS.^11^ UKPDS‐OM2 is a probabilistic, discrete‐time, illness‐death simulation model consisting of 13 linked equations that estimate the likelihood of future macrovascular, microvascular, and fatal events in people with type 2 diabetes. These equations are evaluated in annual cycles in random order to determine the likelihood of events or death occurring. Model equations, based on a median of 17.6 years follow‐up with up to 89 760 patient‐years of data, are internally valid over 25 years.

The model is designed to assess the total burden of disease over an extrapolated lifetime for populations with type 2 diabetes by simulating a range of diabetes‐related complications, life expectancy, quality of life, and relative costs associated with treatment for diabetes‐related complications. It uses a wide variety of input data, including knowledge of previous clinical events for individuals, and has the ability to incorporate changes in some risk factor values over time. The peer‐reviewed equations and algorithms have been published and are freely available.^8^, ^12^ For the present analyses, the model was set to simulate outcomes for the median trial follow‐up period, with observed and predicted values tabulated and shown as cumulative incidence plots.

### 2.4 Statistical methods

UKPDS‐OM2 simulated outcomes and relative risks (RRs) were compared with observed outcomes and hazard ratios (HRs). Simulated estimates were validated using three methods. First, accuracy was assessed by comparing UKPDS‐OM2 simulated event rates with trial‐observed rates and their 95% confidence intervals (CI). Second, we assessed calibration for primary and secondary outcomes in each study using calibration plots that display overall calibration using the calibration intercept and slope.^13^ The calibration slope target value is 1, with values <1 suggesting model estimates are too extreme (i.e., too high for participants who are at high risk and too low for participants who are at low risk) and values >1 suggesting the opposite (i.e., that risk estimates are too moderate). The calibration intercept target value is 0, with negative values suggesting overestimation and positive values suggesting underestimation. Third, we calculated the Brier score,^14^ which takes into account the outcome of the event predicted as well as the estimate. Brier scores range between 0 and 1, with a score of 0 denoting perfect accuracy and a score of 1 denoting perfect inaccuracy. A Brier score of <0.25 considers the model to be informative. In addition, discrimination was evaluated using Harrell's C‐statistic,^15^ a measure of concordance denoting when two individuals are selected at random, the probability of the model assigning a higher risk estimate to individuals with the outcome of interest, compared with the individuals not experiencing this outcome. A value of 1 displays perfect discrimination, with a score of 0.50 considered equivalent to the toss of a coin.

Participant demographics and baseline data entered into UKPDS‐OM2 were age, sex, race/ethnicity, diabetes duration, body weight, height, smoking status, high‐density lipoprotein (HDL)‐cholesterol, low‐density lipoprotein (LDL)‐cholesterol, systolic blood pressure, HbA_1c_, heart rate, white blood cell count, haemoglobin, estimated glomerular filtration rate (eGFR), presence of atrial fibrillation, peripheral arterial disease, and albuminuria. Prior events were also entered, including ischemic heart disease, heart failure, lower extremity amputation, blindness, kidney failure, MI, stroke, and ulcer. Annualized follow‐up data, where available, were entered for weight, smoking status, HDL‐cholesterol, LDL‐cholesterol, systolic blood pressure, HbA_1c_, atrial fibrillation status, peripheral arterial disease, albuminuria, heart rate, white blood cell count, haemoglobin, and eGFR.

Missing data for HbA_1c_, systolic blood pressure, weight, heart rate, and eGFR were all at frequencies <5% (Table S1, Supporting Information). Where individual participant baseline risk factor data were missing or found to lie outside the prespecified model minimum and maximum limits, they were replaced by the age‐ and sex‐adjusted study population mean. If risk factor data were missing for the whole study, then a population average was applied. HDL‐cholesterol, LDL‐cholesterol, and haemoglobin were missing at higher frequencies (19.8%, 25.6%, and 39.7%, respectively). As these were not considered to be missing at random, stratified imputation was used. Missing key follow‐up data were populated using the risk factor progression equations.^12^

UKPDS‐OM2 considers previous clinical events by entering the number of years since such events occurred, with those taking place <1 year before baseline carrying different costs and quality‐adjusted life years (QALYs). As dates of previous clinical events were not recorded in TECOS or EXSCEL, and costs and QALYs are not part of these analyses, all known historical events were set as having occurred 1 year previously. The current version of UKPDS‐OM2 only calculates the risk of first events for ischemic heart disease or heart failure, meaning those individuals with a history of those events do not contribute to the estimates for those equations.

## 3 RESULTS

### 3.1 Accuracy of TECOS estimates

**FIGURE 1:**
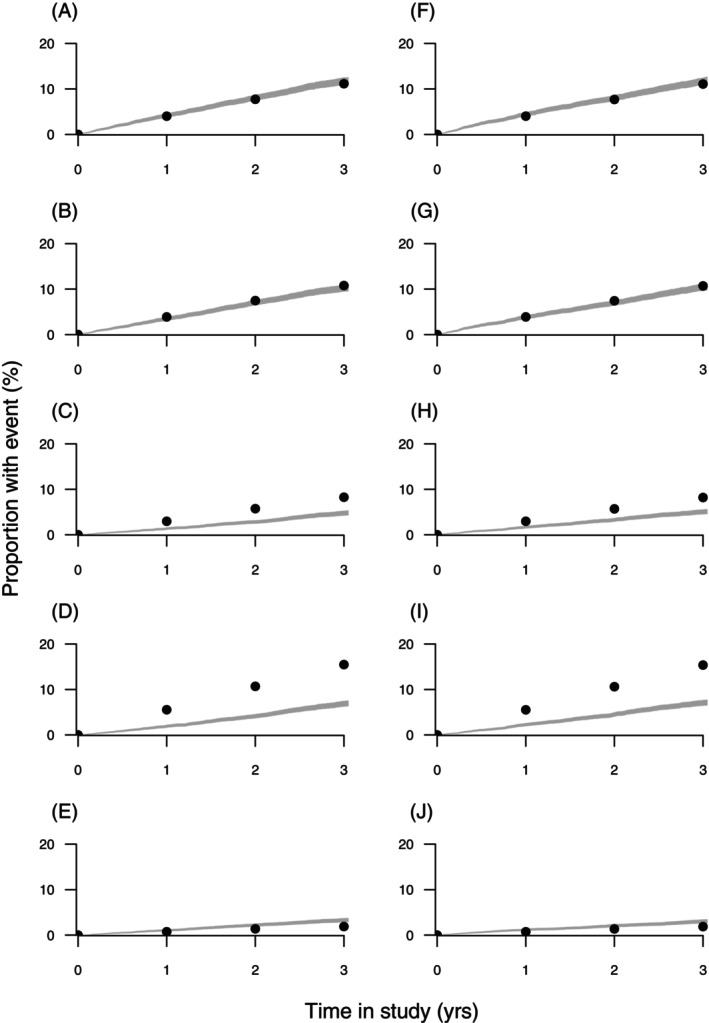
TECOS observed (lines) and estimated (dots) cumulative events for placebo and sitagliptin groups, respectively, for the following outcomes: MACE‐4 (A, F), MACE‐3 (B, G), CV death (C, H), ACM (D, I), and hHF (E, J).

During a median 3.0 (interquartile range 2.3–3.8) years of follow‐up, the primary MACE‐4 composite outcome occurred in 839 (11.4%) and 851 (11.6%) sitagliptin and placebo group participants, respectively (HR 0.98, 95% CI 0.89–1.08). Corresponding UKPDS‐OM2 3‐year simulated outcomes were slightly lower at 776 (10.6%) and 778 (10.6%), respectively, with an RR of 1.00 (Table 1). The MACE‐3 composite outcome occurred in 745 (10.2%) and 746 (10.2%) sitagliptin and placebo group participants, respectively (HR 0.99, 95% CI 0.89–1.10). Corresponding UKPDS‐OM2 3‐year simulated outcomes were again slightly lower at 686 (9.4%) and 687 (9.4%), with an RR of 1.00 (Table 1). Model performance for the individual secondary outcomes underestimated hHF and overestimated CV death, hUA, and ACM (Table 1 and Figure 1). However, simulated between‐treatment‐group RRs were all similar to the HRs seen in the trial.

**TABLE 1:** Observed and simulated events in the TECOS sitagliptin and placebo arms with their corresponding hazard ratios and relative risks.

Outcome	Sitagliptin events (*n* = 7332)		Placebo events (*n* = 7339)		Observed hazard ratio (95%CI)	Simulated relative risk	C‐statistic
	Observed	Simulated	Observed	Simulated			
Primary composite outcome (MACE‐4)	839 (11.4%)	776 (10.6%)	851 (11.6%)	778 (10.6%)	0.98 (0.89–1.08)	1.00	0.62
Secondary composite outcome (MACE‐3)	745 (10.2%)	686 (9.4%)	746 (10.2%)	687 (9.4%)	0.99 (0.89–1.10)	1.00	0.62
Secondary outcomes							
Cardiovascular death	380 (5.2%)	527 (7.2%)	366 (5.0%)	527 (7.2%)	1.03 (0.89–1.19)	1.00	0.65
Fatal or nonfatal myocardial infarction	300 (4.1%)	422 (5.7%)	316 (4.3%)	423 (5.8%)	0.95 (0.81–1.11)	1.00	0.56
Fatal or nonfatal stroke	178 (2.4%)	205 (2.8%)	183 (2.5%)	206 (2.8%)	0.97 (0.79–1.19)	1.00	0.66
All‐cause mortality	547 (7.5%)	1127 (15.4%)	537 (7.3%)	1136 (15.5%)	1.01 (0.90–1.14)	1.00	0.67
Hospitalization for heart failure^a^	131 (2.2%)	84 (1.4%)	135 (2.3%)	83 (1.4%)	0.95 (0.75–1.21)	1.00	0.67
Hospitalization for heart failure or cardiovascular death	538 (7.3%)	651 (8.9%)	525 (8.9%)	656 (7.1%)	1.02 (0.90–1.15)	1.00	0.65

Abbreviations: MACE‐3, 3‐point major adverse cardiovascular event; MACE‐4, 4‐point major adverse cardiovascular event.

^a^ In those without prior heart failure.

### 3.2 Accuracy of EXSCEL estimates

**FIGURE 2:**
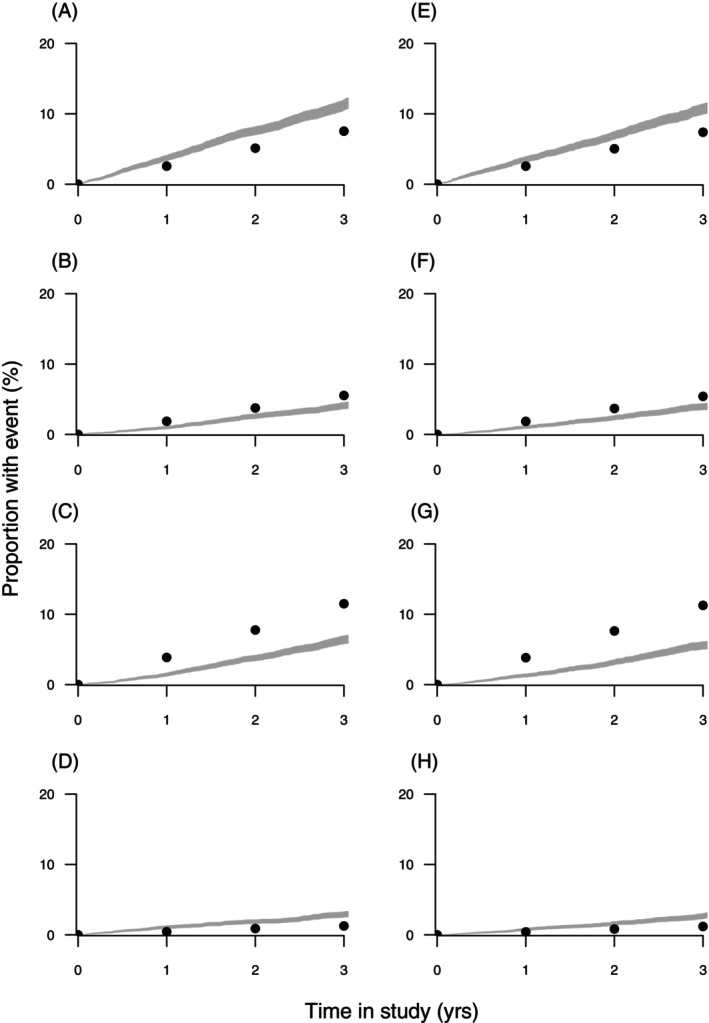
EXSCEL observed (lines) and estimated (dots) cumulative events for placebo and EQW groups, respectively, for the following outcomes: MACE‐3 (A, E), CV death (B, F), ACM (C, G), and hHF (D, H).

During a median 3.2 (2.2–4.4) years of follow‐up, the primary MACE‐3 composite outcome occurred in 839 (11.4%) and 905 (12.2%) EQW and placebo group participants, respectively (HR 0.92, 95% CI 0.84–1.01). Corresponding UKPDS‐OM2 3‐year simulated outcomes were about one‐third lower at 579 (7.9%) and 593 (8.0%), respectively, with a slightly higher RR of 0.98 (Table 2). Model performance for the individual secondary outcomes was similar to that seen in TECOS, with underestimated hHF and overestimated CV deaths and ACM (Table 2 and Figure 2). The largest overestimates were for fatal MI and fatal stroke. Simulated between‐treatment‐group RRs were all similar to the HRs observed in the trial.

**TABLE 2:** Observed and simulated events in the EXSCEL exenatide and placebo arms with their corresponding hazard ratios and relative risks.

Outcome	Exenatide events (*n* = 7356)		Placebo events (*n* = 7396)		Unadjusted hazard ratio (95%CI)	Simulated relative risk	C‐statistic
	Observed	Simulated	Observed	Simulated			
Primary outcome (MACE‐3)	839 (11.4%)	579 (7.9%)	905 (12.2%)	593 (8.0%)	0.92 (0.84–1.01)	0.98	0.68
Secondary outcomes							
All‐cause mortality	507 (6.9%)	883 (12.0%)	584 (7.9%)	906 (12.2%)	0.87 (0.77–0.98)	0.98	0.72
Cardiovascular death	340 (4.6%)	424 (5.8%)	383 (5.2%)	437 (5.9%)	0.89 (0.77–1.03)	0.98	0.71
Fatal or nonfatal myocardial infarction	483 (6.6%)	368 (5.0%)	493 (6.7%)	377 (5.1%)	0.98 (0.86–1.11)	0.98	0.63
Fatal myocardial infarction	17 (0.2%)	206 (2.8%)	13 (0.2%)	211 (2.9%)	1.53 (0.76–3.07)	0.98	0.67
Fatal or nonfatal stroke	187 (2.5%)	167 (2.3%)	218 (2.9%)	175 (2.4%)	0.85 (0.70–1.04)	0.96	0.66
Fatal stroke	18 (0.2%)	77 (1.0%)	25 (0.3%)	79 (1.1%)	0.82 (0.48–1.41)	0.98	0.70
Hospitalization for heart failure^a^	129 (2.1%)	79 (1.3%)	144 (2.3%)	83 (1.3%)	0.88 (0.70–1.12)	0.94	0.72
Hospitalization for acute coronary syndrome	602 (8.2%)	523 (7.1%)	570 (7.7%)	538 (7.3%)	1.06 (0.94–1.19)	0.98	0.63

Abbreviation: MACE‐3, 3‐point major adverse cardiovascular event.

^a^ In those without prior heart failure.

### 3.3 Sensitivity analyses

To determine whether the model performed consistently across demographic groups, the primary outcome model performance was examined by age and sex, and in EXSCEL only, by ASCVD history categories. Tables S2 and S3 show that simulated outcomes were numerically closer to those observed for age <65 years compared with age ≥65 years in TECOS and EXSCEL, although a 2‐fold overestimate for ACM was seen in both trials. Simulated outcomes for both males and females were consistent with those reported overall in both TECOS and EXSCEL (Tables S4 and S5). In EXSCEL, simulated outcomes in those with no prior CV events were numerically closer to those observed than those estimated in individuals with prior CV events (Table S6).

### 3.4 Calibration

The calibration plots displaying observed and simulated rates for TECOS (Figure S1) show an overestimation of events in the highest decile of risk observed during the study for all outcomes except the MACE‐4, MACE‐3, and hHF endpoints. This appears to be largely driven by fatal events, which are overestimated in all except the first decile of risk for death from any cause.

The calibration plots for EXSCEL (Figure S2) also show an overestimation of events in the highest decile of risk observed during the study for death from any cause, CV death, fatal MI and fatal stroke, with an underestimation of events in the highest deciles for the primary MACE composite, hHF, and hACS.

Brier scores for TECOS and EXSCEL (Tables S7 and S8) ranged between 0.02–0.10 and 0.004–0.10, respectively, meaning model results are informative for all outcomes.

### 3.5 Discrimination

In TECOS, the model performed moderately to adequately for all outcomes, with C‐statistic values between 0.62 and 0.67, with the exception of MI for which discrimination performance was poor (0.56) (Table 1). In EXSCEL, the model performed adequately for all outcomes, with C‐statistics between 0.63 and 0.72 (Table 2).

## 4 DISCUSSION

External validation of risk calculation tools is essential to demonstrate the generalizability of their use.^16^ UKPDS‐OM2 was developed using data from individuals aged 25–65 years with type 2 diabetes newly diagnosed between 1977 and 1991, few of whom had a history of CV disease, with up to 30 years of follow‐up.^8^ Assessing the performance of UKPDS‐OM2 using TECOS and EXSCEL data gives insight into its ability to perform in different populations, and specifically those with a longer duration of diabetes and those with or at high risk for ASCVD. Our results show mixed performance, specifically for those with a longer duration of diabetes and those with or at high risk for ASCVD.

Simulated estimates for the primary MACE‐4 composite outcome in TECOS were slightly lower than those observed, compared with underestimates of about one third for the risk for the primary MACE‐3 composite outcome in EXSCEL. Simulated estimates of fatal events in both trials were overestimated, and to a greater degree in TECOS. This may in part be explained by the greater age and prior history of ASCVD for all participants compared with those in EXSCEL. Interestingly, MI and stroke event estimations were closer to those observed, suggesting the model adequately identifies the risk of CV events occurring but does not incorporate the increased survival from such events compared with that historically expected. Of particular note were the UKPDS‐OM2 overestimates for the secondary outcomes of fatal MI and stroke in the EXSCEL trial similar to those seen previously for ASCEND (A Study of Cardiovascular Events in Diabetes) trial data.^17^ The poor discrimination of the model for ischemic heart disease events in TECOS and heart failure events in EXSCEL may be explained in part by how the model defines these compared with definitions used in the studies.

We note the limitation of handling missing data using stratified imputation. As data missing for HDL‐cholesterol, LDL‐cholesterol, and haemoglobin were not considered to be missing at random, the methods used do not eliminate the risk of artificially increasing accuracy. However, given that our results highlight the need for model improvement in these populations, we do not believe this affects the conclusions of this work.

An important potential use of a validated UKPDS‐OM2 is in the design of future clinical outcome trials by performing simulations using the characteristics of the population of interest. UKPDS‐OM2 could be used to estimate the likely impact of the intervention(s) chosen, as demonstrated in this paper, and to aid prediction of likely effect size(s) to help inform sample size calculations.

In conclusion, UKPDS‐OM2 accurately simulated relative risks between randomized groups in both trials. The proportion of participants with the primary outcome was accurately estimated in both TECOS arms but underestimated by around one third in both EXSCEL arms. Overall, these results demonstrate the limitations of models to generalize their use outside the populations from which they were created, in this instance individuals over 65 years with prior CV disease. Evaluation of recalibrated equations is needed to ensure reliable risk estimation for individuals with type 2 diabetes and prior CV disease.

## AUTHOR CONTRIBUTIONS

R.L.C. and R.R.H. were involved in the conception, design, and conduct of the study. R.L.C. conducted the analyses and wrote the first draft of the manuscript. All authors interpreted the results and edited, reviewed, and approved the final version of the manuscript. R.R.H. is the guarantor of this work and, as such, had full access to all the data in the study and takes responsibility for the integrity of the data and the accuracy of the data analysis.

## FUNDING INFORMATION

The TECOS trial was funded by Merck Sharp & Dohme LLC (MSD), a subsidiary of Merck & Co., Inc. (Rahway, NJ). The EXSCEL trial was sponsored and funded by Amylin Pharmaceuticals Inc. (San Diego, CA), a wholly owned subsidiary of AstraZeneca (Gaithersburg, MD). A.I.A. and R.L.C. are supported by the National Institute for Health Research Oxford Biomedical Research Centre.

## CONFLICT OF INTEREST STATEMENT

A.I.A. reports research funding from Novo Nordisk and Sava. D.K.M. reports support for Clinical Trials Leadership from Boehringer Ingelheim, Pfizer, AstraZeneca, Novo Nordisk, Esperion, Lilly USA, CSL Behring, and New Amsterdam; honoraria for consultancy from Lilly USA, Pfizer, Boehringer Ingelheim, Lexicon, Novo Nordisk, Applied Therapeutics, Altimmune, CSL Behring, Bayer, Intercept, and Esperion. R.R.H. reports personal fees from AstraZeneca, Lilly, Merck KGaA, and Novartis. The remaining authors report no disclosures.

## Supporting information


**Data S1.** Supporting Information.

## Data Availability

Summary level and deidentified datasets analysed during the current study are available from the corresponding author on reasonable request, and with approval from established parent clinical trials and cohort study committees.
