# Supplementary material for: Estimating the risk of cardiovascular outcomes and all‐cause mortality in individuals with type 2 diabetes: Validation of the UKPDS outcomes model using TECOS and EXSCEL data
Source: Diabetes Obes Metab. 2025 Nov 12;28(2):1068–75. doi: 10.1111/dom.70280 (PMC12803570; doi:10.1111/dom.70280)
Supplement: Supplementary file 1 — Data S1. Supporting Information. [file DOM-28-1068-s001.docx]

**Table S1:** Baseline characteristics of TECOS and EXSCEL participants showing degree of missingness with complete case and imputed values.

|  | **TECOS** | | | **EXSCEL** | | |
| --- | --- | --- | --- | --- | --- | --- |
| **Category** | **Complete case** | **Imputed** | **Missingness** | **Complete case** | **Imputed** | **Missingness** |
| Age (years) | 66 (8) | 66 (8) | 320 (2.2%) | 62 (9) | 62 (9) | 0 (0.0%) |
| Female sex | 4927 (33.6%) | 4927 (33.6%) | 0 (0.0%) | 4927 (33.6%) | 5603 (38.0%) | 0 (0.0%) |
| Race |  |  |  |  |  |  |
| White/Other | 10959 (75%) | 10959 (75%) |  | 12422 (84%) | 12422 (84%) |  |
| Afro Caribbean | 447 (3%) | 447 (3%) |  | 872 (6%) | 872 (6%) |  |
| Asian Indian | 3265 (22%) | 3265 (22% | 0 (0.0%) | 1452 (10%) | 1452 (10%) | 0 (0.0%) |
| Diabetes duration (years) | 11 (8) | 11 (8) | 12 (0.08%) | 12 (8) | 12 (8) | 0 (0.0%) |
| Weight (kg) | 85 (19) | 85 (19) | 72 (0.5%) | 92 (21) | 92 (21) | 0 (0.0%) |
| Height (m) | 1.67 (0.10) | 1.67 (0.10) | 126 (0.9%) | 1.68 (0.10) | 1.68 (0.10) | 136 (0.9%) |
| HDL-cholesterol (mmol/L) | 1.13 (0.31) | 1.13 (0.29) | 2911 (19.8%) | 1.13 (0.34) | 1.13 (0.31) | 2635 (17.9%) |
| LDL-cholesterol (mmol/L) | 2.29 (0.86) | 2.35 (0.84) | 3750 (25.6%) | 2.38 (0.91) | 2.39 (0.80) | 3441 (23.3%) |
| Systolic blood pressure (mmHg) | 135 (17) | 135 (17) | 42 (0.3%) | 135 (17) | 135 (17) | 42 (0.3%) |
| HbA_1c_ (%) | 7.3 (0.7) | 7.3 (0.7) | 608 (4.1%) | 8.1 (1.0) | 8.1 (1.0) | 0 (0.0%) |
| Heart rate (bpm) | 72 (11) | 72 (11) | 211 (1.4%) | 73 (11) | 73 (11) | 211 (1.4%) |
| White blood cell count (×10^9^/L) | NA | 8 (0) | 14671 (100%) | NA | 8 (0) | 14752 (100%) |
| Hemoglobin (g/L) | 13.6 (1.6) | 13.7 (1.3) | 5037 (34.3%) | 13.8 (1.5) | 13.8 (1.3) | 5851 (39.7%) |
| eGFR (mL/min/1.73 m^2^) | 75 (21) | 75 (21) | 119 (0.8%) | 78 (24) | 78 (24) | 7 (0.05%) |
| Atrial fibrillation (yes/no) | 1167 (8) | 1167 (8) | 0 (0.0%) | 999 (7) | 999 (7) | 0 (0.0%) |
| Peripheral vascular disease (yes/no) | 1433 (17) | 1433 (17) | 0 (0.0%) | 2800 (19) | 2800 (19) | 0 (0.0%) |
| Current smoker (yes/no) | 1678 (11) | 1678 (11) | 0 (0.0%) | 1721 (12) | 1721 (12) | 0 (0.0%) |
| Albuminuria (yes/no) | 2361 (16) | 2361 (16) | 0 (0.0%) | 928 (6) | 928 (6) | 0 (0.0%) |

Data shown are mean (SD) or n (%).

**Table S2:** Age-stratified observed and simulated events in the TECOS sitagliptin and placebo arms with their corresponding hazard ratios and relative risks.

| **Outcomes for age <65 years** | **Sitagliptin events (n=3,302)** | | | **Placebo events (n=3,381)** | | | **Observed Hazard Ratio (95% CI)** | **Simulated Relative Risk** | **C-statistic** |
| --- | --- | --- | --- | --- | --- | --- | --- | --- | --- |
|  | **Observed** | **Simulated** | | **Observed** | **Simulated** | |  |  |  |
| Primary composite outcome (MACE-4) | 293 (8.8%) | 286 (8.6%) | | 300 (9.1%) | 284 (8.6%) | | 0.95 (0.81–1.12) | 0.99 | 0.61 |
| Secondary composite outcome (MACE-3) | 259 (7.8%) | 242 (7.3%) | | 253 (7.7%) | 241 (7.3%) | | 1.00 (0.84–1.19) | 0.99 | 0.60 |
| Secondary outcomes |  |  | |  |  | |  |  |  |
| Cardiovascular death | 122 (3.7%) | 159 (4.8%) | | 106 (3.2%) | 160 (4.9%) | | 1.13 (0.87–1.47) | 0.99 | 0.64 |
| Fatal or nonfatal myocardial infarction | 106 (3.2%) | 172 (5.2%) | | 123 (3.7%) | 172 (5.2%) | | 0.85 (0.65–1.10) | 0.99 | 0.56 |
| Fatal or nonfatal stroke | 66 (2.0%) | 56 (1.7%) | | 64 (1.9%) | 55 (1.7%) | | 1.00 (0.72–1.42) | 0.99 | 0.70 |
| All-cause mortality | 155 (4.7%) | 298 (9.0%) | | 150 (4.5%) | 301 (9.2%) | | 1.01 (0.81–1.27) | 0.99 | 0.63 |
| Hospitalization for heart failure† | 42 (1.5%) | 24 (0.8%) | | 32 (1.1%) | 24 (0.9%) | | 1.28 (0.81–2.03) | 1.00 | 0.65 |
| Hospitalization for heart failure or cardiovascular death | 168 (5.1%) | 176 (5.3%) | | 153 (4.6%) | 177 (5.4%) | | 1.08 (0.86–1.34) | 0.99 | 0.56 |
| **Outcomes for age ≥65 years** | **Sitagliptin events (n=4,030)** | | | **Placebo events (n=4,058)** | | | **Observed Hazard Ratio (95% CI)** | **Simulated Relative Risk** | **C-statistic** |
|  | **Observed** | | **Simulated** | **Observed** | | **Simulated** |  |  |  |
| Primary composite outcome (MACE-4) | 546 (13.6%) | 496 (12.2%) | | 551 (13.7%) | 389 (12.2%) | | 0.99 (0.88–1.12) | 1.00 | 0.60 |
| Secondary composite outcome (MACE-3) | 486 (12.1%) | 449 (11.0%) | | 746 (10.2%) | 493 (12.2%) | | 0.99 (0.87–1.12) | 1.00 | 0.59 |
| Secondary outcomes |  |  | |  |  | |  |  |  |
| Cardiovascular death | 258 (6.4%) | 371 (9.1%) | | 260 (6.4%) | 369 (9.1%) | | 1.00 (0.84–1.18) | 1.00 | 0.61 |
| Fatal or nonfatal myocardial infarction | 194 (4.8%) | 253 (6.2%) | | 193 (4.8%) | 250 (6.2%) | | 1.00 (0.83–1.23) | 1.00 | 0.56 |
| Fatal or nonfatal stroke | 112 (2.8%) | 152 (3.7%) | | 119 (3.0%) | 151 (3.7%) | | 0.94 (0.73–1.22) | 0.99 | 0.63 |
| All-cause mortality | 392 (9.8%) | 802 (19.8%) | | 387 (9.6%) | 792 (19.6%) | | 1.02 (0.88–1.17) | 1.01 | 0.63 |
| Hospitalization for heart failure† | 228 (3.1%) | 59 (1.8%) | | 229 (3.1%) | 59 (1.8%) | | 0.86 (0.65–1.14) | 1.01 | 0.63 |
| Hospitalization for heart failure or cardiovascular death | 370 (9.2%) | 405 (10.0%) | | 372 (9.2%) | 402 (10.0%) | | 0.99 (0.86–1.15) | 1.00 | 0.58 |

† Of participants with no prior HF.

**Table S3:** Age-stratified observed and simulated events in the EXSCEL exenatide and placebo arms with their corresponding hazard ratios and relative risks.

| **Outcomes for age <65 years** | **Exenatide events (n=4,932)** | | | **Placebo events (n=4,421)** | | | **Unadjusted Hazard Ratio (95% CI)** | **Simulated Relative Risk** | **C-statistic** |
| --- | --- | --- | --- | --- | --- | --- | --- | --- | --- |
|  | **Observed** | **Simulated** | | **Observed** | **Simulated** | |  |  |  |
| Primary composite outcome (MACE-3) | 413 (9.4%) | 290 (6.6%) | | 393 (8.9%) | 296 (6.7%) | | 1.05 (0.92–1.21) | 0.99 | 0.69 |
| Secondary outcomes |  |  | |  |  | |  |  |  |
| All-cause mortality | 219 (5.0%) | 359 (8.2%) | | 216 (4.9%) | 368 (8.3%) | | 1.02 (0.84–1.22) | 0.98 | 0.71 |
| Cardiovascular death | 157 (3.6%) | 187 (4.2%) | | 150 (3.4%) | 192 (4.3%) | | 1.05 (0.84–1.31) | 0.98 | 0.73 |
| Fatal or nonfatal myocardial infarction | 220 (5.0%) | 206 (4.7%) | | 209 (4.7%) | 209 (4.7%) | | 0.87 (0.75–1.01) | 0.99 | 0.66 |
| Fatal myocardial infarction | 10 (0.2%) | 99 (2.2%) | | 4 (0.1%) | 102 (2.3%) | | 2.49 (0.78–7.94) | 0.98 | 0.70 |
| Fatal or nonfatal stroke | 103 (2.3%) | 69 (1.6%) | | 101 (2.3%) | 72 (1.6%) | | 1.03 (0.81–1.31) | 0.97 | 0.68 |
| Fatal stroke | 10 (0.2%) | 24 (0.5%) | | 8 (0.2%) | 25 (0.6%) | | 1.24 (0.49–3.14) | 0.96 | 0.74 |
| Hospitalization for heart failure† | 52 (1.4%) | 40 (0.9%) | | 58 (1.6%) | 41 (0.9%) | | 1.12 (0.68–1.83) | 0.98 | 0.72 |
| Hospitalization for acute coronary syndrome | 297 (6.7%) | 253 (5.8%) | | 254 (5.7%) | 260 (5.9%) | | 1.18 (1.00–1.39) | 0.98 | 0.63 |
| **Outcomes for age ≥65 years** | **Exenatide events (n=2,964)** | | | **Placebo events (n=2,975)** | | | **Unadjusted Hazard Ratio (95% CI)** | **Simulated Relative Risk** | **C-statistic** |
|  | **Observed** | | **Simulated** | **Observed** | | **Simulated** |  |  |  |
| Primary composite outcome (MACE-3) | 426 (14.4%) | 321 (10.8%) | | 512 (17.2%) | 333 (11.2%) | | 0.82 (0.72–0.93) | 0.97 | 0.63 |
| Secondary outcomes |  |  | |  |  | |  |  |  |
| All-cause mortality | 288 (9.7%) | 572 (19.3%) | | 368 (12.4%) | 588 (19.7%) | | 0.78 (0.67–0.91) | 0.98 | 0.68 |
| Cardiovascular death | 183 (6.2%) | 265 (8.9%) | | 233 (7.8%) | 274 (9.2%) | | 0.78 (0.65–0.95) | 0.97 | 0.65 |
| Fatal or nonfatal myocardial infarction | 263 (8.9%) | 184 (6.2%) | | 320 (10.8%) | 191 (6.4%) | | 1.06 (0.90–1.25) | 0.97 | 0.57 |
| Fatal myocardial infarction | 10 (0.3%) | 121 (4.1%) | | 9 (0.3%) | 126 (4.2%) | | 1.10 (0.45–2.71) | 0.97 | 0.59 |
| Fatal or nonfatal stroke | 84 (2.8%) | 108 (3.6%) | | 117 (3.9%) | 113 (3.8%) | | 1.12 (0.84–1.48) | 0.96 | 0.62 |
| Fatal stroke | 14 (0.5%) | 57 (2.0%) | | 21 (0.7%) | 59 (2.0%) | | 0.66 (0.34–1.30) | 0.97 | 0.55 |
| Hospitalization for heart failure† | 77 (3.2%) | 59 (2.0%) | | 86 (3.6%) | 65 (2.2%) | | 0.91 (0.69–1.19) | 0.91 | 0.67 |
| Hospitalization for acute coronary syndrome | 305 (10.3%) | 303 (10.2%) | | 316 (10.6%) | 313 (10.5%) | | 0.96 (0.82–1.12) | 0.97 | 0.59 |

† Of participants with no prior HF.

**Table S4:** Sex-stratified observed and simulated events in the TECOS sitagliptin and placebo arms with their corresponding hazard ratios and relative risks.

| **Outcomes for females** | **Sitagliptin events (n=2,134)** | | | **Placebo events (n=2,163)** | | | **Observed Hazard Ratio (95% CI)** | **Simulated Relative Risk** | **C-statistic** |
| --- | --- | --- | --- | --- | --- | --- | --- | --- | --- |
|  | **Observed** | **Simulated** | | **Observed** | **Simulated** | |  |  |  |
| Primary composite outcome (MACE-4) | 203 (9.5%) | 196 (9.1%) | | 215 (9.9%) | 194 (11.1%) | | 0.94 (0.78–1.14) | 1.00 | 0.58 |
| Secondary composite outcome (MACE-3) | 180 (8.2%) | 177 (8.2%) | | 191 (8.8%) | 175 (10.8%) | | 0.94 (0.77–1.15) | 1.00 | 0.58 |
| Secondary outcomes |  |  | |  |  | |  |  |  |
| Cardiovascular death | 105 (4.9%) | 140 (6.5%) | | 94 (5.0%) | 138 (6.4%) | | 1.11 (0.84–1.47) | 1.00 | 0.61 |
| Fatal or nonfatal myocardial infarction | 60 (2.8%) | 107 (4.9%) | | 76 (3.5%) | 107 (5.0%) | | 1.16 (0.69–1.94) | 0.98 | 0.61 |
| Fatal or nonfatal stroke | 47 (2.2%) | 59 (2.7%) | | 49 (2.3%) | 56 (2.6%) | | 0.79 (0.56–1.19) | 1.03 | 0.61 |
| All-cause mortality | 139 (6.5%) | 320 (14.8%) | | 140 (6.5%) | 311 (14.5%) | | 0.99 (0.78–1.25) | 1.02 | 0.65 |
| Hospitalization for heart failure† | 33 (2.0%) | 25 (1.5%) | | 30 (1.8%) | 25 (1.4%) | | 1.12 (0.68–1.83) | 1.04 | 0.63 |
| Hospitalization for heart failure or cardiovascular death | 143 (6.7%) | 157 (7.2%) | | 134 (6.2%) | 153 (7.1%) | | 1.07 (0.84–1.35) | 1.01 | 0.57 |
| **Outcomes for males** | **Sitagliptin events (n=5,198)** | | | **Placebo events (n=5,176)** | | | **Observed Hazard Ratio (95% CI)** | **Simulated Relative Risk** | **C-statistic** |
|  | **Observed** | | **Simulated** | **Observed** | | **Simulated** |  |  |  |
| Primary composite outcome (MACE-4) | 636 (12.3%) | 581 (11.2%) | | 636 (12.2%) | 582 (11.2%) | | 0.98 (0.89–1.08) | 1.00 | 0.62 |
| Secondary composite outcome (MACE-3) | 565 (10.9%) | 510 (9.8%) | | 555 (10.7%) | 511 (9.8%) | | 0.99 (0.89–1.10) | 1.00 | 0.63 |
| Secondary outcomes |  |  | |  |  | |  |  |  |
| Cardiovascular death | 275 (5.3%) | 387 (7.5%) | | 272 (5.3%) | 390 (7.5%) | | 1.03 (0.89–1.19) | 1.00 | 0.66 |
| Fatal or nonfatal myocardial infarction | 240 (4.1%) | 316 (6.1%) | | 240 (4.6%) | 315 (6.1%) | | 0.95 (0.81–1.11) | 1.00 | 0.54 |
| Fatal or nonfatal stroke | 131 (2.4%) | 147 (2.8%) | | 134 (2.6%) | 149 (2.8%) | | 0.97 (0.79–1.19) | 1.00 | 0.68 |
| All-cause mortality | 408 (7.5%) | 779 (15.0%) | | 397 (7.7%) | 785 (15.1%) | | 1.01 (0.90–1.14) | 1.00 | 0.67 |
| Hospitalization for heart failure† | 98 (2.2%) | 58 (1.3%) | | 105 (2.4%) | 60 (1.4%) | | 0.91 (0.67–1.19) | 0.98 | 0.69 |
| Hospitalization for heart failure or cardiovascular death | 395 (7.6%) | 421 (8.1%) | | 391 (7.6%) | 425 (8.2%) | | 1.02 (0.90–1.15) | 1.00 | 0.62 |

† Of participants with no prior HF.

**Table S5:** Sex-stratified observed and simulated events in the EXSCEL exenatide and placebo arms with their corresponding hazard ratios and relative risks.

| **Outcomes for females** | **Exenatide events (n=2,794)** | | | **Placebo events (n=2,809)** | | | **Unadjusted Hazard Ratio (95% CI)** | **Simulated Relative Risk** | **C-statistic** |
| --- | --- | --- | --- | --- | --- | --- | --- | --- | --- |
|  | **Observed** | **Simulated** | | **Observed** | **Simulated** | |  |  |  |
| Primary composite outcome (MACE-3) | 240 (8.6%) | 178 (6.4%) | | 271 (7.4%) | 185 (6.6%) | | 0.87 (0.73–1.04) | 0.97 | 0.69 |
| Secondary outcomes |  |  | |  |  | |  |  |  |
| All-cause mortality | 142 (5.1%) | 299 (10.7%) | | 179 (6.4%) | 307 (10.9%) | | 0.79 (0.64–0.99) | 0.98 | 0.73 |
| Cardiovascular death | 97 (3.5%) | 132 (4.7%) | | 115 (4.1%) | 137 (4.9%) | | 0.84 (0.64–1.10) | 0.97 | 0.73 |
| Fatal or nonfatal myocardial infarction | 123 (4.4%) | 115 (4.1%) | | 127 (4.5%) | 119 (4.2%) | | 0.96 (0.75–1.22) | 0.97 | 0.66 |
| Fatal myocardial infarction | 5 (0.2%) | 67 (2.4%) | | 5 (0.5%) | 69 (2.4%) | | 0.99 (0.29–3.43) | 0.97 | 0.78 |
| Fatal or nonfatal stroke | 68 (2.4%) | 55 (1.9%) | | 80 (2.8%) | 58 (2.0%) | | 0.84 (0.61–1.16) | 0.96 | 0.68 |
| Fatal stroke | 13 (0.5%) | 26 (0.9%) | | 9 (0.3%) | 27 (0.9%) | | 1.44 (0.62–3.36) | 0.96 | 0.70 |
| Hospitalization for heart failure† | 40 (1.7%) | 38 (1.3%) | | 44 (1.9%) | 41 (1.4%) | | 0.88 (0.57–1.35) | 0.92 | 0.75 |
| Hospitalization for acute coronary syndrome | 165 (5.9%) | 161 (5.8%) | | 147 (5.2%) | 168 (6.0%) | | 1.11 (0.89–1.39) | 0.96 | 0.64 |
| **Outcomes for males** | **Exenatide events (n=4,562)** | | | **Placebo events (n=4,587)** | | | **Unadjusted Hazard Ratio (95% CI)** | **Simulated Relative Risk** | **C-statistic** |
|  | **Observed** | | **Simulated** | **Observed** | | **Simulated** |  |  |  |
| Primary composite outcome (MACE-3) | 599 (13.1%) | 431 (9.4%) | | 634 (13.8%) | 441 (9.6%) | | 0.94 (0.84–1.05) | 0.98 | 0.66 |
| Secondary outcomes |  |  | |  |  | |  |  |  |
| All-cause mortality | 365 (8.0%) | 633 (13.9%) | | 405 (8.8%) | 650 (14.2%) | | 0.90 (0.78–1.04) | 0.98 | 0.71 |
| Cardiovascular death | 243 (5.3%) | 318 (7.0%) | | 268 (5.8%) | 326 (7.1%) | | 0.91 (0.76–1.08) | 0.98 | 0.70 |
| Fatal or nonfatal myocardial infarction | 360 (7.9%) | 274 (6.0%) | | 366 (8.0%) | 281 (6.1%) | | 0.98 (0.85–1.14) | 0.98 | 0.59 |
| Fatal myocardial infarction | 15 (0.3%) | 153 (3.3%) | | 8 (0.2%) | 157 (3.4%) | | 1.86 (0.79–4.38) | 0.98 | 0.61 |
| Fatal or nonfatal stroke | 119 (2.6%) | 120 (2.6%) | | 138 (3.0%) | 126 (2.7%) | | 0.86 (0.67–1.10) | 0.96 | 0.65 |
| Fatal stroke | 11 (0.2%) | 55 (1.2%) | | 20 (0.4%) | 57 (1.2%) | | 0.54 (0.26–1.14) | 0.97 | 0.71 |
| Hospitalization for heart failure† | 89 (2.3%) | 61 (1.3%) | | 100 (2.6%) | 64 (1.4%) | | 0.88 (0.66–1.17) | 0.96 | 0.70 |
| Hospitalization for acute coronary syndrome | 437 (9.6%) | 393 (8.6%) | | 423 (9.2%) | 402 (8.7%) | | 1.04 (0.91–1.19) | 0.98 | 0.61 |

† Of participants with no prior HF.

**Table S6:** Prior ASCVD–stratified observed and simulated events in the EXSCEL exenatide and placebo arms with their corresponding hazard ratios and relative risks.

| **Outcomes for no prior CV event** | **Exenatide events (n=1,962)** | | | **Placebo events (n=2,008)** | | | **Unadjusted Hazard Ratio (95% CI)** | **Simulated Relative Risk** | **C-statistic** |
| --- | --- | --- | --- | --- | --- | --- | --- | --- | --- |
|  | **Observed** | | **Simulated** | **Observed** | **Simulated** | |  |  |  |
| Primary composite outcome (MACE-3) | 117 (6.0%) | | 82 (4.2%) | 119 (5.9%) | 89 (4.4%) | | 0.99 (0.77–1.28) | 0.95 | 0.66 |
| Secondary outcomes |  | |  |  |  | |  |  |  |
| All-cause mortality | 71 (3.6%) | | 115 (5.8%) | 93 (4.6%) | 125 (6.2%) | | 0.77 (0.57–1.05) | 0.94 | 0.72 |
| Cardiovascular death | 43 (2.2%) | | 48 (2.4%) | 53 (2.6%) | 52 (2.6%) | | 0.82 (0.55–1.23) | 0.94 | 0.75 |
| Fatal or nonfatal myocardial infarction | 59 (3.0%) | | 53 (2.7%) | 53 (2.6%) | 57 (2.8%) | | 1.13 (0.78–1.63) | 0.96 | 0.62 |
| Fatal myocardial infarction | 0 (0.0%) | | 25 (1.2%) | 3 (0.1%) | 26 (1.3%) | | - | 0.96 | 0.82 |
| Fatal or nonfatal stroke | 32 (1.6%) | | 25 (1.3%) | 33 (1.6%) | 27 (1.3%) | | 0.97 (0.60–1.58) | 0.95 | 0.63 |
| Fatal stroke | 4 (0.2%) | | 8 (0.4%) | 2 (0.1%) | 9 (0.4%) | | 2.02 (0.37–11.0) | 0.93 | 0.72 |
| Hospitalization for heart failure† | 19 (1.1%) | | 21 (1.1%) | 23 (1.3%) | 23 (1.1%) | | 0.82 (0.45–1.51) | 0.95 | 0.81 |
| Hospitalization for acute coronary syndrome | 71 (3.6%) | | 84 (4.3%) | 58 (2.9%) | 90 (4.4%) | | 1.25 (0.88–1.76) | 0.96 | 0.66 |
| **Outcomes for prior CV event** | **Exenatide events (n=5,394)** | | | **Placebo events (n=5,388)** | | | **Unadjusted Hazard Ratio (95% CI)** | **Simulated Relative Risk** | **C-statistic** |
|  | **Observed** | **Simulated** | | **Observed** | | **Simulated** |  |  |  |
| Primary composite outcome (MACE-3) | 722 (13.4%) | | 528 (9.8%) | 786 (14.6%) | 538 (10.0%) | | 0.90 (0.82–1.00) | 0.98 | 0.65 |
| Secondary outcomes |  | |  |  |  | |  |  |  |
| All-cause mortality | 436 (8.1%) | | 768 (14.2%) | 491 (9.1%) | 781 (14.5%) | | 0.88 (0.77–1.00) | 0.98 | 0.71 |
| Cardiovascular death | 297 (5.5%) | | 378 (7.0%) | 330 (6.1%) | 386 (7.2%) | | 0.89 (0.76–1.05) | 0.98 | 0.68 |
| Fatal or nonfatal myocardial infarction | 424 (7.9%) | | 317 (5.9%) | 440 (8.2%) | 322 (6.0%) | | 0.95 (0.83–1.09) | 0.98 | 0.58 |
| Fatal myocardial infarction | 20 (0.4%) | | 182 (3.4%) | 10 (0.2%) | 186 (3.4%) | | 1.97 (0.92–4.20) | 0.98 | 0.61 |
| Fatal or nonfatal stroke | 155 (2.9%) | | 143 (2.6%) | 185 (3.4%) | 148 (2.7%) | | 0.82 (0.67–1.02) | 0.96 | 0.65 |
| Fatal stroke | 20 (0.4%) | | 70 (1.3%) | 27 (0.5%) | 71 (1.3%) | | 0.73 (0.41–1.30) | 0.98 | 0.66 |
| Hospitalization for heart failure† | 110 (2.5%) | | 59 (1.3%) | 121 (2.8%) | 62 (1.4%) | | 0.89 (0.69–1.15) | 0.94 | 0.69 |
| Hospitalization for acute coronary syndrome | 531 (9.8%) | | 472 (8.7%) | 512 (9.5%) | 481 (8.9%) | | 1.03 (0.91–1.16) | 0.98 | 0.58 |

† Of participants with no prior HF.

**Figure S1:** Calibration plots for primary and secondary outcomes in TECOS. Observed event proportions (circle) with 95% confidence intervals (vertical line) by decile of risk during the study. The dashed line represents perfect correlation.

**
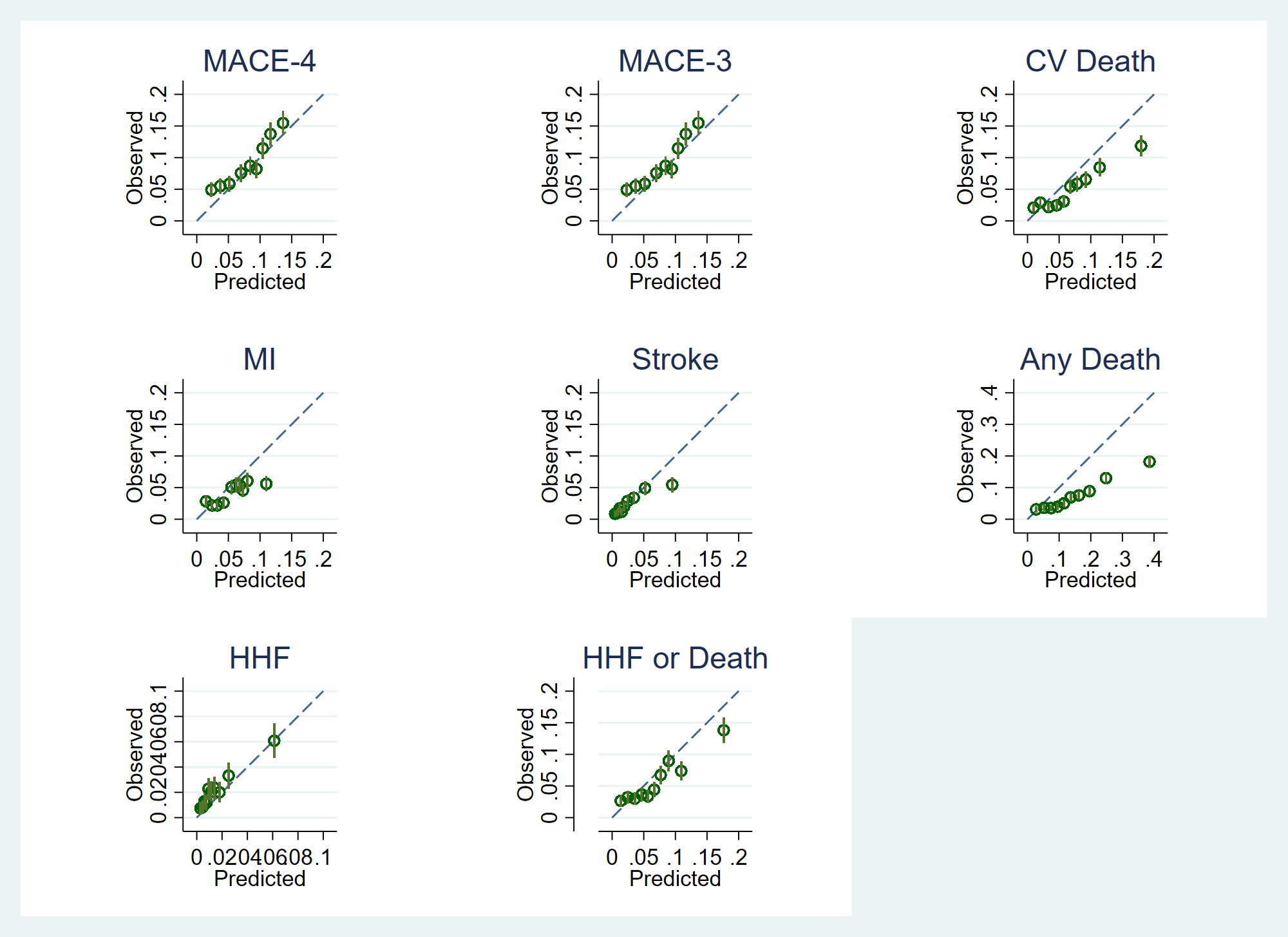
**

**Figure S2:** Calibration plots for primary and secondary outcomes in EXSCEL. Observed event proportions (circle) with 95% confidence intervals (vertical line) by decile of risk during the study. The dashed line represents perfect correlation.

**
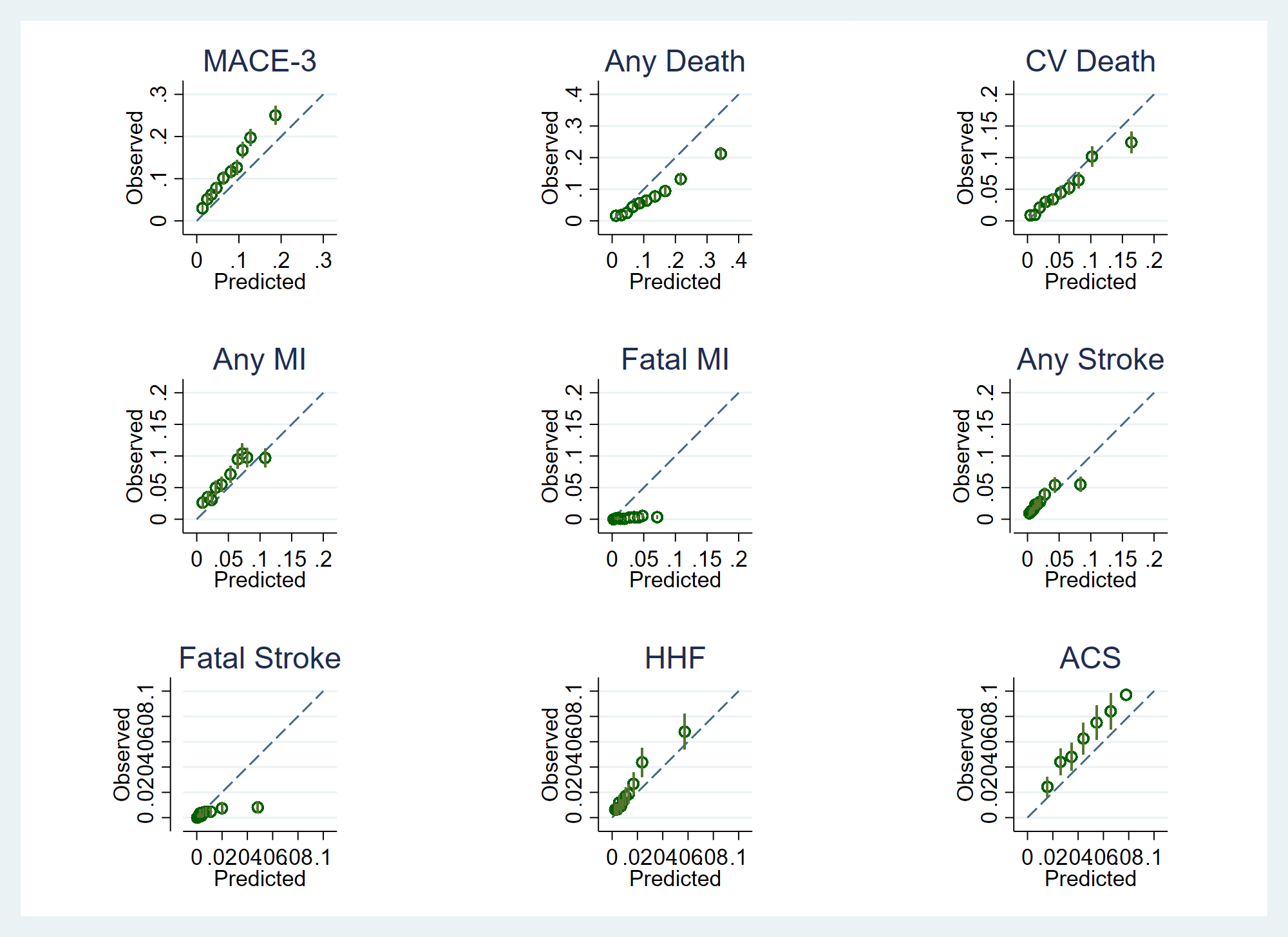
**

**Table S7:** Brier scores for primary and secondary events in TECOS.

| **Event type** | **Brier Score** |
| --- | --- |
| MACE-4 | 0.10 |
| MACE-3 | 0.09 |
| Cardiovascular death | 0.05 |
| Fatal or nonfatal myocardial infarction | 0.04 |
| Fatal or nonfatal stroke | 0.02 |
| All-cause mortality | 0.07 |
| Hospitalization for heart failure | 0.02 |
| Hospitalization for heart failure or cardiovascular death | 0.05 |

**Table S8:** Brier scores for primary and secondary events in EXSCEL.

| **Event type** | **Brier Score** |
| --- | --- |
| MACE-3 | 0.10 |
| All-cause mortality | 0.07 |
| Cardiovascular death | 0.05 |
| Fatal or nonfatal myocardial infarction | 0.06 |
| Fatal myocardial infarction | 0.003 |
| Fatal or nonfatal stroke | 0.03 |
| Fatal stroke | 0.004 |
| Hospitalization for heart failure | 0.02 |
| Hospitalization for acute coronary syndrome | 0.07 |
